# Variation in prostate cancer growth rates in an MRI-based active surveillance cohort

**DOI:** 10.1007/s00330-025-12248-y

**Published:** 2026-01-16

**Authors:** Hayley Smith, Vasilis Stavrinides, Francesco Giganti, Caroline M. Moore, Bharath Narayanan, Mark Emberton, Paul D. P. Pharoah, Nora Pashayan

**Affiliations:** 1 Department of Public Health and Primary Care, University of Cambridge, Cambridge, UK; 2 UCL Cancer Institute, University College London, London, UK; 3 Department of Urology, University College London Hospitals NHS Trust, London, UK; 4 Department of Imaging, Imperial College Healthcare, London, UK; 5 Division of Surgery and Interventional Science, University College London, London, UK; 6 Department of Radiology, University College London Hospital NHS Foundation Trust, London, UK; 7 Faculty of Medical Sciences, University College London, London, UK; 8 Cedars-Sinai Medical Center, Los Angeles, CA USA

**Keywords:** Prostate cancer, Magnetic resonance imaging, Tumour growth modelling, Active surveillance, Early detection of cancer

## Abstract

**Background:**

Understanding tumour growth rates helps optimise screening and active surveillance (AS) schedules. We estimated prostate cancer growth rate accounting for individual variation in a longitudinal AS cohort.

**Materials and methods:**

We modelled tumour growth in 145 biopsy-confirmed prostate cancer patients undergoing MRI-based AS. Primary lesion volumes were measured longitudinally using planimetry. We compared three mixed-effects models (exponential, Gompertz, and logistic) and investigated relationships between growth rate and clinical characteristics. We estimated the natural trajectory of prostate cancer lesions starting at a single cell to clinical detectability (diameter ≈ 1 cm), with diameters estimated based on spherical volume.

**Results:**

All three models fit observed data well; however, only the Gompertz model provided reasonable estimates from a single cell to an MRI-detectable size (diameter ≈ 3 mm). The Gompertz growth parameter (mean = 0.07, range = 0.02–0.15), describing exponential growth deceleration, was positively correlated with: patient age; lesion volume at AS onset; prostate-specific antigen (PSA) level; and PSA density. Lesions with Gleason 3 + 4 had faster volume doubling times than Gleason 3 + 3 lesions (mean = 3.5 and 5.2 years, respectively). On average, it would take 17 years (95% CI [15, 19]) for a lesion to grow from a single cell to an MRI-detectable size and an additional 12 years to reach a clinically detectable size (95% CI [10, 13]). At age 50, 75% of lesions would remain undetectable by MRI.

**Conclusions:**

Prostate cancer shows slow growth with large variation between patients, posing a challenge for early detection.

**Key Points:**

***Question*** *What is the population distribution of growth rates and the natural history of prostate cancer?*

***Findings*** *Prostate cancer typically grows slowly, with considerable variation between individuals. On average, lesions take 17 years to grow from initiation to an MRI-detectable size.*

***Clinical relevance*** *Small lesions undetectable on MRI may take many years to reach a clinically significant size, posing a challenge for early detection, as it increases the risk of detecting indolent lesions that may never cause harm.*

**Graphical Abstract:**

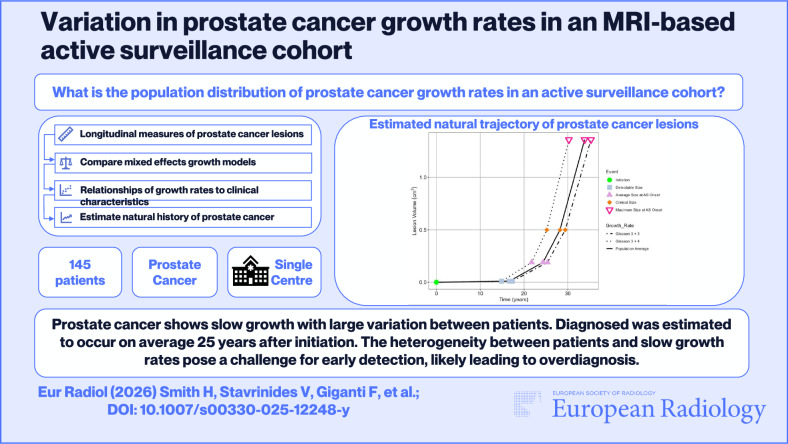

## Introduction

Prostate cancer (PCa) is the second most common cancer in men worldwide, with over 1.4 million new cases and 400,000 deaths in 2022 [1]. The European Randomised Study of Screening for PCa showed over a 20% reduction in cancer mortality, but at the cost of overdiagnosis and overtreatment in many others [2, 3].

Active surveillance (AS) reduces overtreatment in PCa by deferring treatment until there is evidence of cancer progression for low- to intermediate-risk PCa, i.e., Gleason 3 + 3 and 3 + 4 cancers, which are often slow growing, without losing the window of curability [4]. There is no standardised protocol for AS, though patients are frequently seen annually. Due to differences in progression rates between patients, additional appointments could lead to unnecessary burden for patients and the healthcare system [5].

Using tumour growth rates, we can understand the biological behaviour of PCa and tailor AS time intervals to capture meaningful progression as early as possible, while keeping appointments to a minimum. Tumour growth rates based on PSA change have previously been used as biomarkers of progression during treatment in prostate cancer [6, 7]. We can also use growth rates to identify optimal screening schedules.

Previous studies have quantified the average growth of PCa using measurements of tumour volume at two time points [8, 9] or PSA levels as a proxy for tumour size [10, 11]. However, these studies assume continuous growth over time and only quantify average growth, ignoring how growth may change over time. Tumour growth can vary between patients with the same cancer type [12–14] and within the same patient over time [15, 16]. It is likely that tumour growth decelerates as the tumour grows larger [17–20] and may grow in a stepwise manner rather than consistently [15].

There are many spatial-temporal and cell-dynamic based models to quantify tumour growth, as discussed in Jørgensen et al in 2023 [21]. These models typically require multimodal imaging and histology data. In this study, we have a unique dataset of longitudinal volumes based on MRI for patients not receiving active treatment; hence, we use mixed-effects longitudinal growth models to estimate the population distribution of growth rates.

We aim to characterise the natural history of PCa by modelling the population distribution of growth and identify factors associated with faster-growing disease.

## Materials and methods

### Active surveillance dataset

We followed the STROBE checklist for cohort studies to ensure transparent and complete reporting [22].

Institutional Review Board approval was not required for our analysis. Approval was not required for the AS cohort because at the institution at which the data was collected, all clinical records and MR images are routinely reviewed as part of an audit performed for the internal evaluation of the AS service as per UK NICE guidelines.

This was a retrospective cohort study using existing data. The cohort comprised 553 patients enrolled in our MR-led AS programme at University College London Hospital (London, UK) from 2005 to 2020 [23]. Patients had biopsy-confirmed low- to intermediate-risk PCa according to National Institute for Health and Care Excellence guidelines (i.e., Gleason ≤ 3 + 4 and baseline PSA ≤ 20 ng/mL) [24], and at least two multiparametric MRIs performed between December 2005 and January 2020. Clinical data were available, including results from biopsies performed prior to and/or during AS.

Tumour volumes (cm^3^) were estimated from MRI scans [8]. A dedicated genitourinary radiologist (FG) identified for each patient the index lesion using all scans to ensure consistency and calculated lesion volumes by planimetry measured dimensions on all sequences (T2-Weighted imaging, Diffusion-Weighted Imaging, and Dynamic Contrast Enhancement imaging). The MRI and lesion measurement protocol has previously been published [8]. The radiologist assigned the Prostate Cancer Radiologic Estimation of Change in Sequential Evaluation (PRECISE) score, quantifying radiologic change over time for each follow-up scan [25]. A PRECISE score of 1 or 2 indicates radiological regression, a PRECISE score of 3 entails radiological stability, and a PRECISE score of 4 or 5 implies radiological progression.

**Fig. 1:**
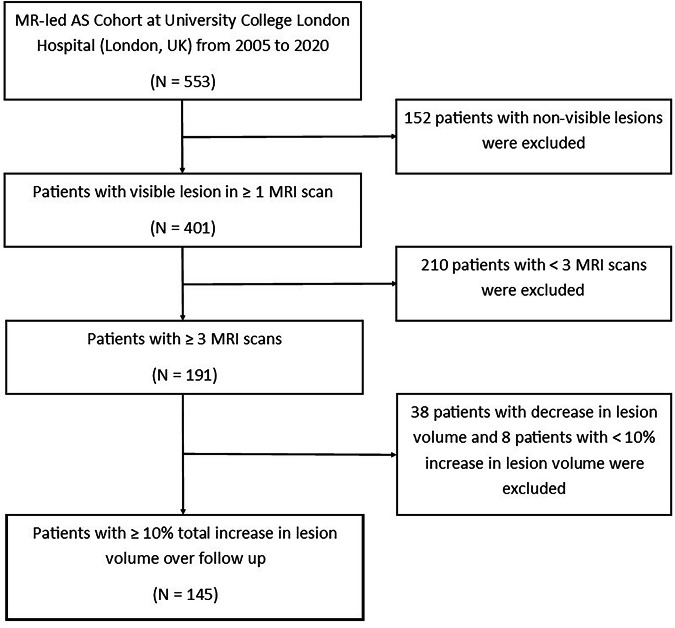
Eligibility and inclusion flowchart. 145 patients were included in our analysis. The inclusion criteria were: (1) visible prostate cancer lesion; (2) ≥ 3 MRI scans at distinct time points; and (3) ≥ 10% increase in lesion volume over follow-up

For our study, we included patients who had a visible lesion, ≥ 3 planimetric lesion measurements at distinct time points, and ≥ 10% total volume increase over the follow-up period (*N* = 145), allowing us to evaluate three different growth models. We excluded patients with < 10% total volume increase as changes below this threshold over the follow-up period are likely to be MRI or measurement variability, not true growth [26]. Figure 1 shows the eligibility and inclusion flowchart.

### Describing lesion growth

We calculated average percentage change per year assuming exponential growth:

1 $$\% \,{change\; per\; year}=\,\left[{\left(\frac{{V}_{{last}}}{{V}_{{first}}}\right)}^{\frac{1}{{time}}}-1\right]x\,100$$

We also calculated tumour volume doubling time (TVDT), using the exponential growth rate α:

2 $$V\left(t\right)=\,{V}_{0}\exp \left({{\rm{\alpha }}}* t\right)$$

3 $${TVDT}=\,\frac{{\mathrm{ln}}\left(2\right)}{{{\rm{\alpha }}}}$$

### Comparing non-linear mixed-effects models for PCa growth

To estimate PCa growth rates, we must account for the correlations between lesion measurements from the same patient. Mixed-effects models handle this by modelling grouped data, such as repeated measurements per patient. Each patient represents a level in the model, which estimates the population average (fixed effects) and variation within and between individuals (random effects), assuming normality of residuals and random effects. We define time zero as entry to AS.

Cancer growth may follow varying trajectories. The exponential model assumes growth is constant over time and is given by:

4 $${V}_{j}^{i}=\, {V}_{0}^{i}\, {exp}\left({{{\rm{\alpha }}}}^{i}\, \times {time}\right)+\, {\varepsilon }_{j}^{i}$$

where:

5 $${V}_{0}^{i}={\mu }_{V}+\,{\eta }_{V}^{i}$$

6 $${{{\rm{\alpha }}}}^{i}=\,{\mu }_{{{\rm{\alpha }}}}+\,{\eta }_{{{\rm{\alpha }}}}^{i}$$

Here, ${V}_{j}^{i}$ is lesion volume for individual *i* at time *j*. The patient-specific intercept, ${V}_{0}^{i}$, combines the fixed effect ${\mu }_{V}$ and individual random effect ${\eta }_{V}^{i}$. The patient-specific growth parameter ${{{\rm{\alpha }}}}^{i}$ includes the fixed effect ${\mu }_{{{\rm{\alpha }}}}$ and individual random effect ${\eta }_{{{\rm{\alpha }}}}^{i}$. ${\varepsilon }_{j}^{i}$ is the error term for individual *i* at time *j*. In the exponential model, cancer grows indefinitely.

Preclinical and clinical studies show cancer growth slows as resources are depleted [17–20]. The Gompertz model captures this by assuming growth slows exponentially as the tumour reaches some limiting size *K*. Here, $\beta$ is the deceleration parameter—higher values indicate faster slowing as the tumour nears *K*. The Gompertz mixed-effects model is given by:

7 $${V}_{j}^{i}={{\rm{K}}}\exp \left[{{\mathrm{ln}}}\left[\frac{{V}_{0}^{i}}{{{\rm{K}}}}\right]\exp \left( - {\beta }^{i}\,\times {{\rm{time}}}\right)\right] + \, {\varepsilon }_{j}^{i}$$

The logistic model assumes growth slows linearly as the tumour nears *K* and is given by:

8 $${V}_{j}^{i} = \frac{{V}_{0}^{i}{{\rm{K}}}}{{V}_{0}^{i} + \left({{\rm{K }}}-{V}_{0}^{i}\right)\exp \left(-{\beta }^{i} \, \times {{\rm{time}}}\right)} \, + \, {\varepsilon }_{j}^{i}$$

For the Gompertz and logistic models, the patient-specific deceleration parameter ${\beta }^{i}$ includes the fixed effect ${\mu }_{\beta }$ and individual random effect ${\eta }_{\beta }^{i}$:

9 $${\beta }^{i}=\,{\mu }_{\beta }+\,{\eta }_{\beta }^{i}$$

We modelled PCa lesion growth using these three mixed-effects models, assuming continuous growth of the lesions. We modelled log volume so outcomes were normally distributed and transformed the equations accordingly (see Supplementary 2). We also included random effects for intercept and time as their incorporation yielded superior model fits (Likelihood Ratio test: *p* < 0.01). Mixed-effects models assume the random effects and residuals are normally distributed. While the outcomes themselves may not be strictly normal, severe deviations in these underlying distributions can violate model assumptions and impact estimation.

The volume limit *K* in the Gompertz and logistic models were fixed at 10 cm^3^ (three times the maximum volume in the dataset) before parameter estimation. We conducted a sensitivity analysis where the performance of the model and estimated parameters were robust to the choice of *K*, indicating our results hold under different values (see Supplementary 3).

We compared the three different models to assess both their fit to the AS dataset and their ability to extrapolate outside the range of the data. We compared the model fit to the AS dataset using Akaike Information Criterion (AIC), Bayesian Information Criterion (BIC), mean absolute error (MAE), and percentage error (% error) between observed and predicted lesion volumes. We tested performance on the full dataset and validated by splitting into training and testing sets (see Supplementary 4).

We estimated the time from lesion volume equal to a single cell (1e-9 cm^3^) to a detectable size on MRI (0.01 cm^3^) by solving the model equations for *t* (Eqs. 10–12), where V = 0.01 cm^3^ and V_0_ = 1e-9 cm^3^. We assumed continuous, deterministic growth throughout this period; however, it is important to acknowledge that in tumour development, growth may not be strictly deterministic and can be susceptible to periods of dormancy followed by spurts.

*Exponential:*

10 $${{\rm{t}}}=\frac{1}{\alpha }log \left(\frac{V}{{V}_{0}}\right)$$

*Gompertz:*

11 $${{\rm{t}}}=-\frac{1}{\beta }log \left(\frac{\log \left(V\right)-\log (K)}{\log \left({V}_{0}\right)-\log (K)}\right)$$

*Logistic:*

12 $${{\rm{t}}}=-\frac{1}{\beta }\log \left[\left(\frac{{V}_{0}\,\times \,K}{V}-\,{V}_{0}\right)\times \,\frac{1}{K-\,{V}_{0}}\right]$$

### Comparing growth rates with patient and tumour characteristics

We selected the Gompertz model for further evaluations because it presents a more biologically plausible model of tumour growth; this is evidenced by the estimates of the time since disease initiation and the robustness of these estimates to changes in the parameter K.

Using stratified analyses, we compared patient-specific $\beta$ parameters from the Gompertz model with clinical characteristics at diagnosis: patient age; lesion volume; Gleason score; prostate volume; PRECISE score [8, 25]; PSA (ng/mL); and PSA Density (PSAD = PSA/prostate volume). For Gleason and PRECISE scores, we calculated the average $\beta$ for each group (e.g., Gleason 3 + 3 and 3 + 4). Due to the limited number of measurements per patient, it was not feasible to incorporate these variables directly into a multivariate growth model. As such, these stratified analyses were exploratory in nature and do not adjust for potential confounding between clinical variables (e.g. PSA and PSAD are likely correlated with lesion volume and age).

For continuous variables (age, lesion volume, prostate volume, PSA, and PSA density), associations with the estimated deceleration parameters ($\beta$) were assessed using Pearson correlation coefficients. For categorical variables, $\beta$ values were compared between the groups using the Wilcoxon rank-sum test for the Gleason score, given their non-normal distribution per group, and the Welch’s *t*-test for PRECISE score, based on approximate normality and unequal group variances. To facilitate analysis, the PRECISE score was categorised into two groups: ≤ 3 and > 3.

### Estimating the natural trajectory of a PCa lesion

Using the Gompertz model and Equation (11), we estimated when a lesion would reach milestone volumes (diameter based on spherical lesion): initiation (volume = single cell); detectable by MRI (0.01 cm^3^; diameter ≈ 3 mm); average size at AS onset in the dataset (0.19 cm^3^; diameter ≈ 7 mm); clinically detectable (0.5 cm^3^; diameter ≈ 1 cm); and the maximum volume at AS in the dataset (1.36 cm^3^; diameter ≈ 1.4 cm). We estimated sojourn time - the time interval from MRI detectability to clinical detectability.

We selected 0.01 cm^3^ (diameter ≈ 3 mm) as the MRI-detectable size - the smallest volume recorded in the entire AS cohort (*N* = 553). We used a clinically detectable size of 0.5 cm^3^ to approximate a lesion with diameter = 1 cm. This size is detectable on digital rectal examination (DRE) depending on patient anatomy and tumour location [27], and is reported as a threshold volume for determining clinically significant cancers [28].

We estimated the time for lesions to increase by 50% from the starting volume at AS onset; this was agreed by the PRECISE scoring system version 2 panellists to define radiological progression [29]. To estimate when prostate lesions might become detectable, we used our fitted growth model and the patient-specific growth rates to retrospectively predict lesion volumes at age 50 and 60 for each patient. This allowed us to estimate the proportion of patients who would have had MRI-detectable and clinically detectable lesions at those ages, based on volume thresholds of 0.01 cm^3^ and 0.5 cm^3^ respectively.

All analyses were conducted in R v4.4.2 [30] implemented in R Studio [31] using packages: readxl v1.4.3 [32]; dplyr v1.1.4 [33]; ggplot2 [34]; gridExtra v2.3 [35]; lmtest [36]; lme4 v3.1 [37]; and nlme v3.1 [38].

## Results

### Description of the active surveillance dataset

**Fig. 2:**
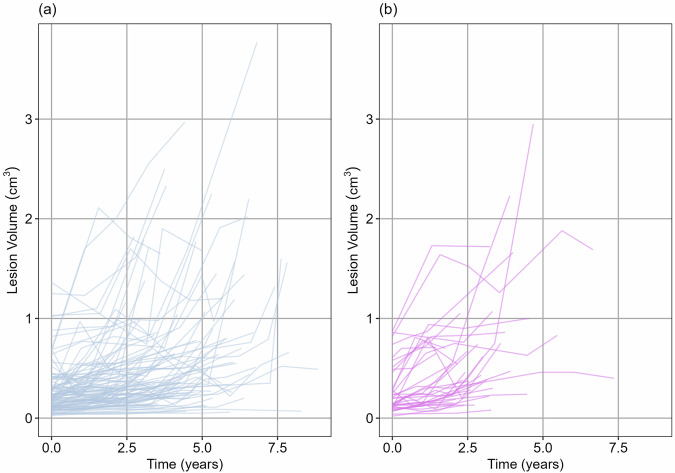
Individual trajectories of lesion volumes (cm^3^) over time for patients with **a** Gleason 3 + 3 at diagnosis, and **b** Gleason 3 + 4 at diagnosis. The volume at first MRI and trajectory slope vary between individual patients

Table 1 shows characteristics of the 145 eligible patients. Figure 2 shows lesion volume trajectories over time. Further cohort details are in Supplementary 1 and Stavrinides et al [23].

**Table 1:** Descriptive statistics of the eligible MRI AS dataset

No. of patients = 145	
Age at diagnosis (years)	62.9 (57.7–67.9)
Follow-up time (years)	3.8 (2.9–5.2)
Time between scans (years)	1.1 (1.0–1.4)
Initial lesion volume (cm^3^)	0.2 (0.1–0.3)
Final lesion volume (cm^3^)	0.5 (0.3–0.9)
All lesion volumes (cm^3^)	0.3 (0.2–0.6)
Prostate volume (cm^3^)	45.6 (34.3–64.1)
PSA (ng/mL)	7.6 (5.3–10.9)
PSAD	0.2 (0.1–0.2)
Number of scans	No.
3	51 (35%)
4	47 (32%)
5	25 (17%)
6	13 (9%)
7	4 (3%)
8	4 (3%)
9	1 (1%)
Initial Gleason score	
3 + 3	108 (74%)
3 + 4	37 (26%)
PRECISE score	
1	5 (3%)
2	3 (2%)
3	9 (6%)
4	108 (74%)
5	20 (14%)

Continuous variables are given as median (interquartile range) to 1 decimal place, and count variables are given as number (%)

*AS* active surveillance, *PSA* prostate-specific antigen, *PSAD* prostate-specific antigen density

### Description of lesion growth

Lesions increased by a median value of 0.3 cm^3^ (122% increase) over the follow-up period and grew 23% per year, with individual patient growth ranging from 2.6% to 175%, assuming a constant growth rate over follow-up. The population average TVDT was 3.1 years. Lesion size at first MRI was negatively correlated with percentage change per year (R = −0.3, *p* < 0.01). Larger lesions at the start of AS had a smaller percentage change per year than smaller lesions; however, this may not reflect the change in absolute size. There was at least one period of inactivity for 53% of patients (defined as the time between two MRI scans during which the lesion volume changed by less than ± 10%), lasting between 2.6 months and 3.3 years (median = 1.1; IQR = 0.97–1.3). A lesion may be inactive between two consecutive scans but then show significant progression at a later scan; since the exact timing of progression between scans is unknown, the measured inactivity intervals only represent the periods confidently observed as stable.

### Comparison of non-linear mixed-effects models for PCa growth

The exponential model had lower AIC and BIC; however, the differences in MAE and % error were negligible (Table 2). The Gompertz model had more reasonable estimates for lesion growth from a single-cell volume to an MRI-detectable size (median = 17.4 years, IQR = 14.1–20.7), compared to the exponential and logistic models (median = 76.4 years, IQR = 51.7–101.1; and median = 65.5 years, IQR = 41.4–89.6, respectively). Supplementary 4 details model calibration, evaluation, and validation.

**Table 2:** Mixed-effects models estimated parameter values (95% confidence intervals) and performance measures to 2 decimal places for the exponential, Gompertz, and logistic models

	Exponential		Gompertz		Logistic	
	Estimate	95% CI	Estimate	95% CI	Estimate	95% CI
Fixed V_0_	−1.62*	−1.76, −1.49*	0.22	0.20, 0.25	0.23	0.20, 0.25
*α*, β	0.22	0.20, 0.25	0.07	0.07, 0.08	0.25	0.23, 0.28
Random V_0_	0.78	0.68, 0.88	0.15	0.12, 0.18	0.15	0.13, 0.18
*α*, β	0.11	0.08, 0.13	0.03	0.03, 0.04	0.11	0.08, 0.14
Correlation	−0.23	−0.44, 0.01	0.44	0.11, 0.66	0.04	−0.27, 0.33
Residual	0.33	0.30, 0.35	0.35	0.33, 0.39	0.35	0.32, 0.38
AIC	946.50		1017.68		1012.87	
BIC	973.01		1044.19		1039.38	
MAE	0.09		0.11		0.11	
% Error	19.85%		21.80%		21.35%	

K (volume limit) is fixed at 10 cm^3^ for the Gompertz and logistic models

V_0_ is the estimated volume at the implementation of AS; α is the estimated growth parameter for the exponential model; and β is the estimated growth deceleration parameter for Gompertz and logistic models

Fixed parameters are the estimated population average; random parameters are the estimated SD of variation within the population

*AIC* Akaike Information Criterion, *BIC* Bayesian Information Criterion, *MAE* mean absolute error, *% error* percentage error, *AS* active surveillance

* Log V_0_ estimate

We selected the Gompertz model for further analysis because performance on the AS data was similar across the models, and the Gompertz model had the most reasonable estimates when extrapolating outside the range of data.

### Relationship between growth rates and patient and tumour characteristics

The Gompertz model had a normally distributed population growth rate with mean = 0.07 per year (SD = 0.03), where patient-specific estimated growth parameters (Eq. 9) ranged from 0.02 to 0.15 per year. Smaller growth parameter values indicate slower growth deceleration, meaning the tumour may grow rapidly for longer. Larger values suggest more aggressive tumours that grow quickly but plateau sooner as they reach the volume limit.

Patient age at diagnosis was positively correlated with estimated growth rate (R = 0.23, *p* < 0.01). Lesion volume, presenting PSA, and PSAD at first MRI were positively correlated with growth rate (R = 0.53, 0.19, and 0.19; *p* < 0.01, = 0.02, and = 0.02). There was no relationship between prostate volume and estimated growth rate. Supplementary 5 shows the correlations between patient and tumour characteristics and growth rate.

**Fig. 3:**
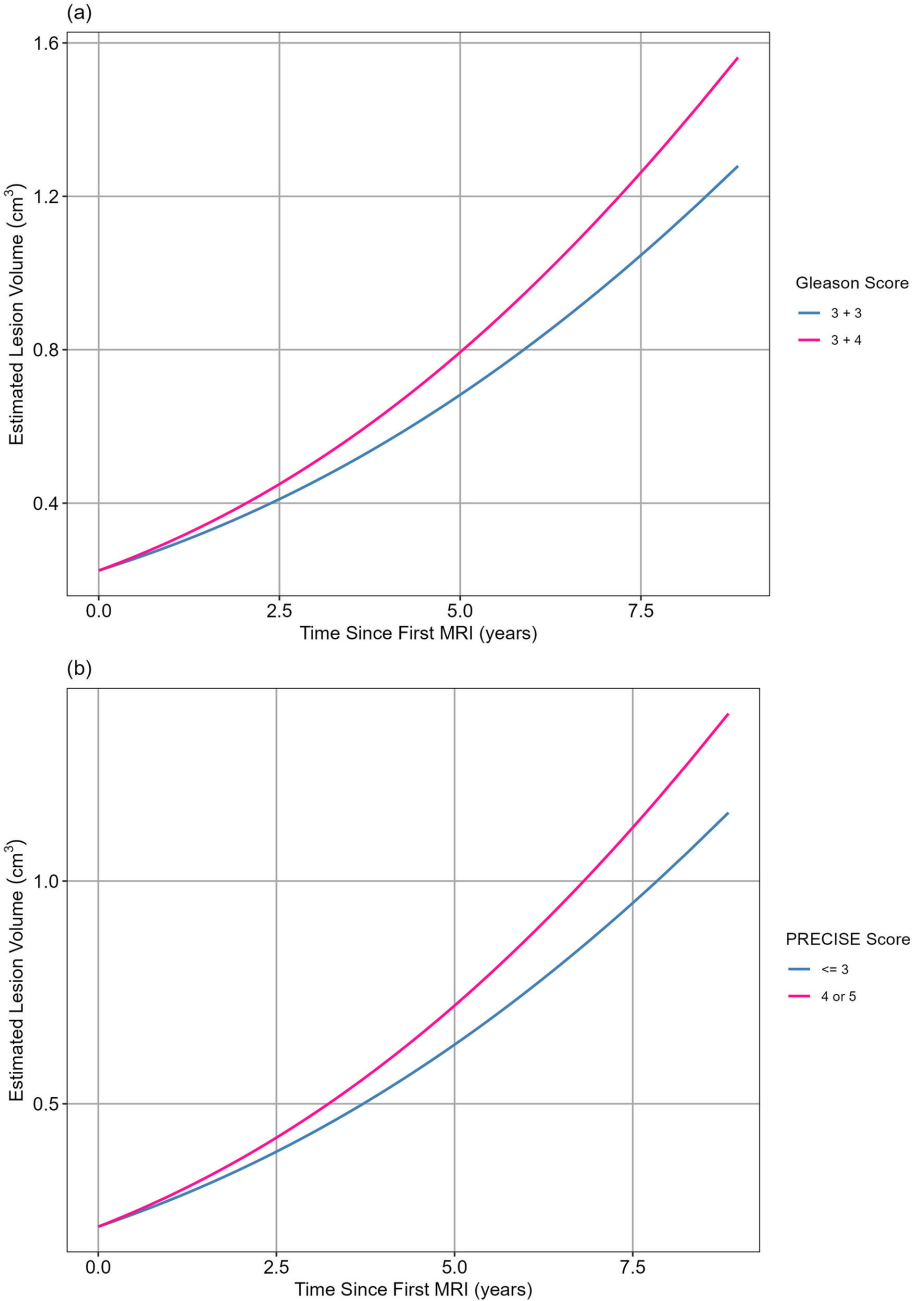
Estimated growth trajectory of lesions with different **a** Gleason score at diagnosis, and **b** PRECISE score. **a** Gleason 3 + 4 lesions have a higher average growth rate than Gleason 3 + 3 lesions. **b** Lesions with an overall PRECISE score of 4 or 5 have faster growth than lesions with a PRECISE score of 1, 2, or 3. The sample size for PRECISE scores 1, 2, 3, and 5 are relatively small

Gleason 3 + 4 lesions at diagnosis had higher growth parameters on average (mean = 0.08) than Gleason 3 + 3 lesions (mean = 0.07), a difference that was statistically significant (Wilcoxon rank-sum test, *p* = 0.009). The mean TVDT (assuming exponential growth) was 5.2 years for Gleason 3 + 3 compared to 3.5 years for Gleason 3 + 4 at diagnosis. Patients with PRECISE score 4 or 5 had faster growth rates (mean = 0.07) than PRECISE score ≤ 3 (mean = 0.06), relating to doubling times of 9 years and 11 years, respectively. This difference was statistically significant (Welch’s *t*-test, *p* = 0.04). Figure 3 shows the estimated growth trajectory of a Gleason 3 + 3 vs. 3 + 4 lesion, and different PRECISE scores.

### Estimated natural trajectory of a PCa lesion

**Fig. 4:**
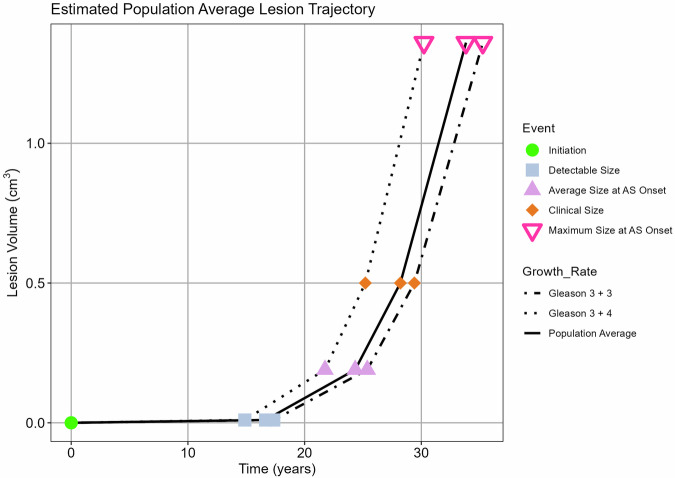
The estimated lesion trajectory from a single cell (1e-9 cm^3^) to the maximum volume at AS onset in the AS dataset (1.36 cm^3^) using three growth rates: the population average (solid line); the average for Gleason 3 + 3 (dot-dash line); and the average for Gleason 3 + 4 (dotted line). The time of key size milestones is plotted: tumour initiation (single cell, circle); detectable size (0.01 cm^3^, square); average size at AS onset (0.19 cm^3^, triangle); clinical size (0.5 cm^3^, diamond); and maximum size at AS onset (1.36 cm^3^, inverted triangle). On average, the lesion will reach an MRI-detectable size in 17 years (17 years for Gleason 3 + 3 lesions and 15 years for Gleason 3 + 4 lesions). It will reach a clinically detectable size by 29 years with a median sojourn time of 12 years. It will reach the maximum volume at AS onset in the AS dataset in 34 years. AS, active surveillance

Figure 4 shows the average trajectory for a PCa lesion from initiation to clinically detectable size.

We estimated a median time of 17 years for a PCa lesion to grow from a single cell (1e-9 cm^3^) to an MRI-detectable volume (volume = 0.01 cm^3^; diameter ≈ 3 mm), ranging from 8 to 51 years for individual patients. While a Gleason 3 + 3 lesion would take 17 years to be MRI detectable, a Gleason 3 + 4 lesion would take 15 years. We estimated that by age 50, 20% of the PCa lesions would be MRI detectable, increasing to 66% by age 60.

We estimated a median time of 29 years to grow from a single cell to a clinically detectable volume, e.g., palpable on DRE (volume = 0.5 cm^3^; diameter ≈ 1 cm), ranging from 13 to 86 years for the individual patients. Gleason 3 + 3 lesions would take 29 years to reach a clinically detectable volume, whereas Gleason 3 + 4 lesions would take 25 years. At age 50, none of the lesions in the dataset would be clinically detectable, and only 15% would be clinically detectable by age 60.

The estimated sojourn time ranged from 5 years to 35 years (median = 12 years). The sojourn time was 12 years for Gleason 3 + 3 and 10 years for Gleason 3 + 4 lesions.

We estimated a median time of 1.6 years (ranging from 8 months to 3.7 years) for a lesion to increase by 50%, deemed a significant change by PRECISE version 2 [29].

## Discussion

This study characterised the growth rate of PCa and factors affecting lesion growth. The Gompertz growth parameter varied greatly between patients, ranging from 0.02 to 0.15 per year. Smaller values indicate tumours that would have a slower deceleration of growth and steadily reach the maximum size, whereas larger values indicate faster initial growth, which slows down rapidly as the lesion reaches the volume limit. Although our cohort is comprised of low- to medium-risk disease, there is still significant variation in growth, demonstrating the substantial differences observed in other cancer types [12–14]. The population average percentage change per year using an exponential model was 25%; however, this ranged from 7% to 62%, showing how the average growth rate in a sample may not reflect the true range of growth rates within a population.

We identified several factors related to lesion growth rate. Older age, larger lesion volume at AS entry, Gleason 3 + 4 at diagnosis, higher PSA, higher PSAD, and PRECISE score 4 or 5 at diagnosis were associated with larger growth parameters. Due to the limited number of MRI time points per patient, these variables could not be included in a multivariate growth model, so any potential confounding between variables (e.g., PSA and lesion volume) cannot be excluded. Further research into the relationship between PSA, PSAD, and growth rate could determine who is at risk of having fast-growing, aggressive PCa at diagnosis and inform treatment options [39–43].

While the Gompertz model assumes continuous smooth growth over time, observation in this cohort suggests lesion growth may occur in a stepwise pattern. We observed periods of lesion stability lasting anywhere from 2 months to 4 years, with some lesions subsequently increasing in volume during continued active surveillance. This raises questions about what causes periods of stability and growth, and how this impacts cancer monitoring and treatment. In this study, we use a simplified continuous growth model to estimate the smoothed average growth over time. While this is useful for summarising long-term growth dynamics, we acknowledge that it may not fully reflect the potentially episodic nature of tumour growth. Detecting lesions that remain stable over time may risk overdiagnosis and overtreatment, particularly for lesions that remain indolent and never progress to clinically significant disease within the patient’s natural lifetime.

We estimated that patients were diagnosed with PCa 25 years after the initiation event on average, with a large range between individual patients. The lesions would take 17 years on average to reach an MRI-detectable size; small Gleason 6 lesions that are not detectable on MRI could remain undetectable for many years. These results reflect estimates from a cell-based study, suggesting prostate cancer likely starts when men are in their 20 s and 30 s, taking 50+ years to progress to metastatic disease [44]. For visible lesions, we expect a 50% increase in lesion volume to occur 1.6 years after AS onset; yearly MRI follow-up may be too frequent to detect meaningful change [9]. Further research on the risk factors associated with faster-growing disease, such as polygenic risk score, with larger sample sizes, could lead to personalised AS protocols, reducing invasive examinations and receiving more timely treatment if progression is likely.

Additionally, the average sojourn time was 12 years from an MRI detectable to a clinical size. Pashayan et al [45] estimated a mean sojourn time of 11.3 to 12.6 years for men aged 50–69 with PCa, in keeping with our estimates. We also estimated that 80% of PCa lesions would not be detectable by MRI at age 50, though at age 60, over 60% would be detectable. However, at age 60, we estimated 85% of lesions would not be detectable by DRE. This is important for future screening studies, as age 50 may be too early to detect most lesions on MRI and could take many years to grow to a clinically significant-sized lesion.

This study provides a new perspective on PCa growth. Using the unique longitudinal AS dataset, we modelled growth in untreated patients who had serial lesion volume measurements over time (up to 9 years). However, these were low- to intermediate-risk patients with visible lesions on MRI and are unrepresentative of the entire PCa population. MRI visibility itself is a risk factor for progression. Our findings may not apply to higher-grade lesions or those that are detectable on biopsy but not by MRI.

We expect some degree of measurement variability in the lesion volumes, even when measured by a single highly experienced radiologist. While planimetry measurements are the most accurate, single diameter measurements are the most reproducible [40]. This inevitable variability may impact the accuracy and robustness of the growth models.

MRI timing varied between patients and may have depended on factors believed to lead to a higher risk of progression. While our mixed-effects model accounts for unequal numbers of scans per patient, there remains a potential for bias if higher-risk patients were scanned more frequently or, conversely, exited active surveillance earlier due to treatment. This could lead to overrepresentation of slower-growing lesions in the dataset and may bias growth rate estimates toward more indolent disease.PCa is frequently slow growing [12]; therefore, a long follow-up period is necessary to observe different phases of PCa growth. We estimated growth based on a short period in relation to the whole tumour trajectory, limiting the reliability of extrapolations beyond the observed data. Although we model back to an assumed initiation point (e.g., a single cell), this estimate is not precise and is only intended to illustrate the extended time it may take for PCa to reach a detectable size.

PCa is often a multi-lesion cancer; however, a single lesion per patient was followed in this analysis; growth may differ between lesions in the same patient. We could not estimate the total volume of cancer within the prostate, which may impact growth and certainly will have a relationship with PSA levels. This is an area of research we were unable to explore in this analysis.

Our findings highlight the significant variation in growth rates among lesions, even within a low- to medium-risk cohort, providing new insights into the progression of this disease. Our estimates suggest that PCa lesions are typically slow growing and could take years to reach a detectable size; small Gleason 6 lesions that are initially undetectable on MRI may require years to reach an MRI-detectable volume. We estimate that most PCa lesions would not be MRI-detectable by age 50, meaning screening strategies could be targeted toward older age groups. The heterogeneity between patients, slow growth rates, and periods of no growth pose a challenge for early detection, likely leading to overdiagnosis.

## Supplementary information

Supplementary information

## Data Availability

The data used in this study are not publicly available, and sharing is not permitted due to containing sensitive patient information.

## Abbreviations

% error: Percentage error
AIC: Akaike Information Criterion
AS: Active surveillance
BIC: Bayesian Information Criterion
DRE: Digital rectal examination
MAE: Mean absolute error
MRI: Magnetic resonance imaging
PCa: Prostate cancer
PRECISE: Prostate cancer radiologic estimation of change in sequential evaluation
PSA: Prostate-specific antigen
PSAD: Prostate-specific antigen density
