# Supplementary material for: Variation in prostate cancer growth rates in an MRI-based active surveillance cohort
Source: Eur Radiol. 2026 Jan 16;36(6):5094–105. doi: 10.1007/s00330-025-12248-y (PMC13212372; doi:10.1007/s00330-025-12248-y)
Supplement: Supplementary file 1 — Supplementary information [file 330_2025_12248_MOESM1_ESM.pdf]

Variation in Prostate Cancer Growth Rates in an MRI-based Active Surveillance Cohort

ELECTRONIC SUPPLEMENTARY MATERIAL

Supplementary 1: Further details of the active surveillance dataset

For the 145 patients with growing lesions, there were 613 volume measurements in total. Patients had between three and nine scans, performed 1.3 years apart on average. Patients were between 48 and 80 years old at the time of diagnosis (mean = 62.6; s.d. = 7.1) and followed up for between 1 and 9 years (mean = 4.1; s.d. = 1.6). Mean PSA level at diagnosis was 8.4ng/ml and mean PSAD was 0.2 ng/ml/cm^3.

Supplementary Figure S1.1 shows histograms of patient age at diagnosis (years); follow-up time (years); initial primary lesion volume (cm^3); primary lesion volumes (cm^3) at all time points; PSA (ng/ml); and PSAD (ng/ml/cm^3).

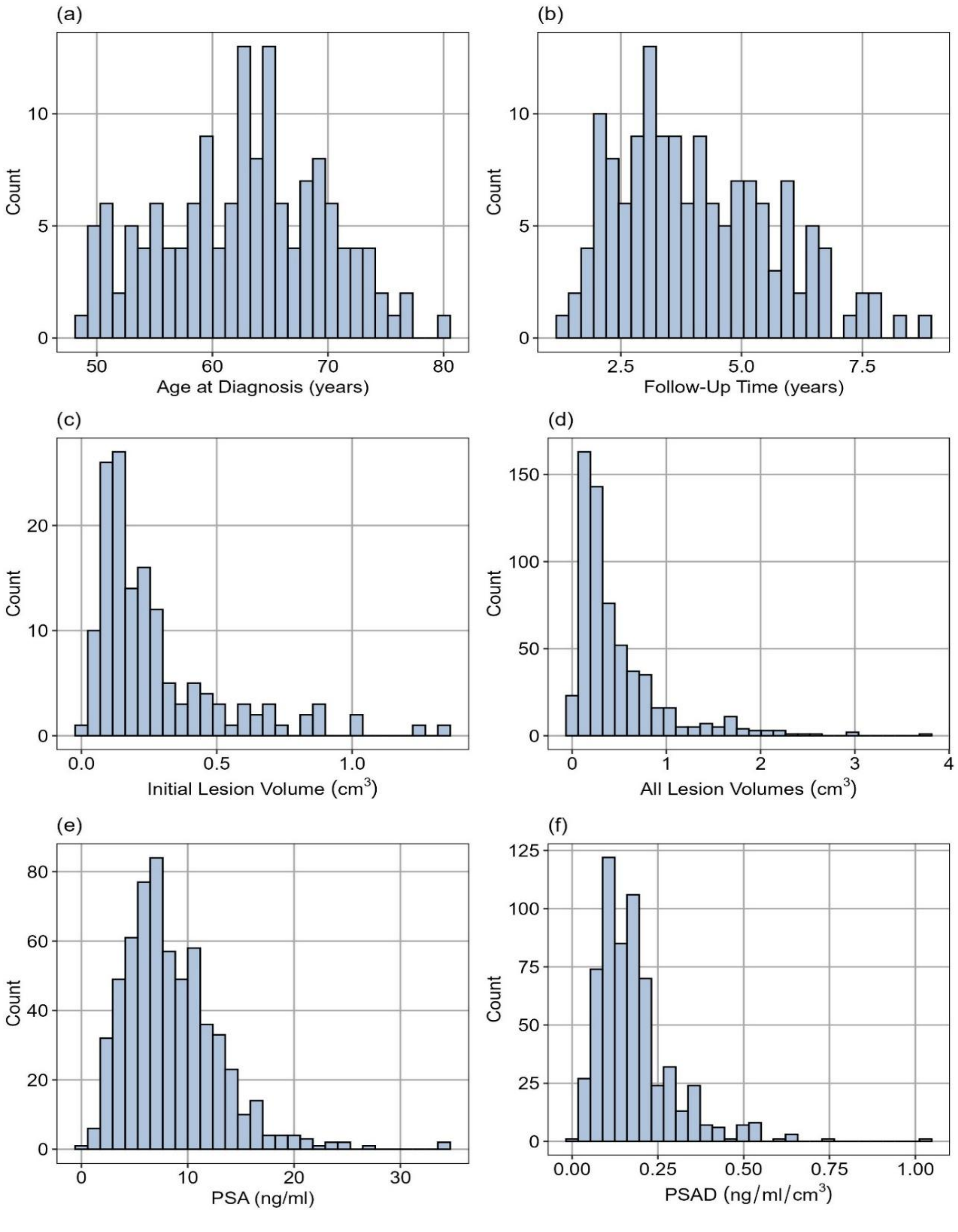

Figure S1.1: Histograms of (a) patient age at diagnosis (years); (b) follow-up time (years); (c) initial primary lesion volume (cm^3); (d) primary lesion volumes (cm^3) at all time points; (e) PSA (ng/ml); and (f) PSAD (ng/ml/cm^3).

Supplementary 2: Log-transformed mixed effects models

We modelled log volume to ensure the outcomes were normally distributed to aid model fitting and transformed the equations accordingly.

V_j^i is lesion volume for individual *i* at time *j*. The patient-specific intercept, V_0^i, combines the fixed effect μ_V and individual random effect η_V^i. The patient-specific growth parameter α^i includes the fixed effect μ_α and individual random effect η_α^i. The patient-specific deceleration parameter β^i includes the fixed effect μ_β and individual random effect η_β^i. ε_j^i is the error term for individual *i* at time *j*.

Exponential model

$$V_j^i = V_0^i \, exp\left(\alpha^i \times time\right) + \varepsilon_j^i \tag{1}$$

$$\ln V_j^i = \ln V_0^i + \alpha^i + \varepsilon_j^i \tag{2}$$

Gompertz model

$$V_j^i = \mathrm{K} \exp\left[\ln\left[\frac{V_0^i}{\mathrm{K}}\right] \exp\left(-\beta^i \times time\right)\right] + \varepsilon_j^i \tag{3}$$

$$\ln V_j^i = \ln K + \ln\left[\frac{V_0^i}{\mathrm{K}}\right] \exp\left(-\beta^i \times time\right) + \varepsilon_j^i \tag{4}$$

Logistic model

$$V_j^i = \frac{V_0^i \mathrm{K}}{V_0^i + \left(\mathrm{K} - V_0^i\right) \exp(-\beta^i \times time)} + \varepsilon_j^i \tag{5}$$

$$\ln V_j^i = \ln V_0^i + \ln K - ln\left[V_0^i + \left(K - V_0^i\right) \exp\left(-\beta^i \times time\right)\right] + \varepsilon_j^i \tag{6}$$

Supplementary 3: Sensitivity analysis for parameter K

We performed a sensitivity analysis of the K parameter for both the Gompertz and logistic mixed effects model. We selected K = 4, …, 12cm^3 to represent values from the largest volume in the dataset (3.77 cm^3) to three times the largest volume.

For each value of K, the Gompertz and logistic models were fit to the dataset to estimate the model parameters: fixed intercept; random intercept; fixed growth rate; and random growth rate (Figure S3.1). There was very little variation in the estimated fixed and random intercept parameter values across values of K for both models (< 2%). This was also true for the logistic model fixed and random growth parameters (< 8%). For the Gompertz fixed and random growth parameters there was 26% and 31% variation in the estimates from K = 4 to K = 12, however, the mean values were 0.08 and 0.04 respectively, indicating a negligible absolute deviation.

We calculated performance measures of the models: Akaike Information Criterion (AIC), Bayesian Information Criterion (BIC), mean absolute error (MAE), and percentage error (% error) between the observed and predicted volumes (Figure S3.2). The AIC and BIC were robust to changes in K for both models. The MAE slightly decreased with larger K values for both models, however, it is within +/- 5% of the mean value. The percent error increased with larger values of K for both models, with a maximum 8% increase for the logistic model.

For both models, there was minimal change in the average estimated time for a lesion to grow from 1 cell volume to an MRI detectable size for K = 4,… , 12 (Figure S3.3). Gompertz estimates were within 17% of the mean (absolute change = 2.6 years) and logistic estimates were within 6% of the mean (absolute change = 3.3 years).

Overall, there was minimal change in the model parameters and the performance of the models with varying values of K. For this analysis, we chose K = 10 as this represents ~ 3x the maximum volume in the dataset.

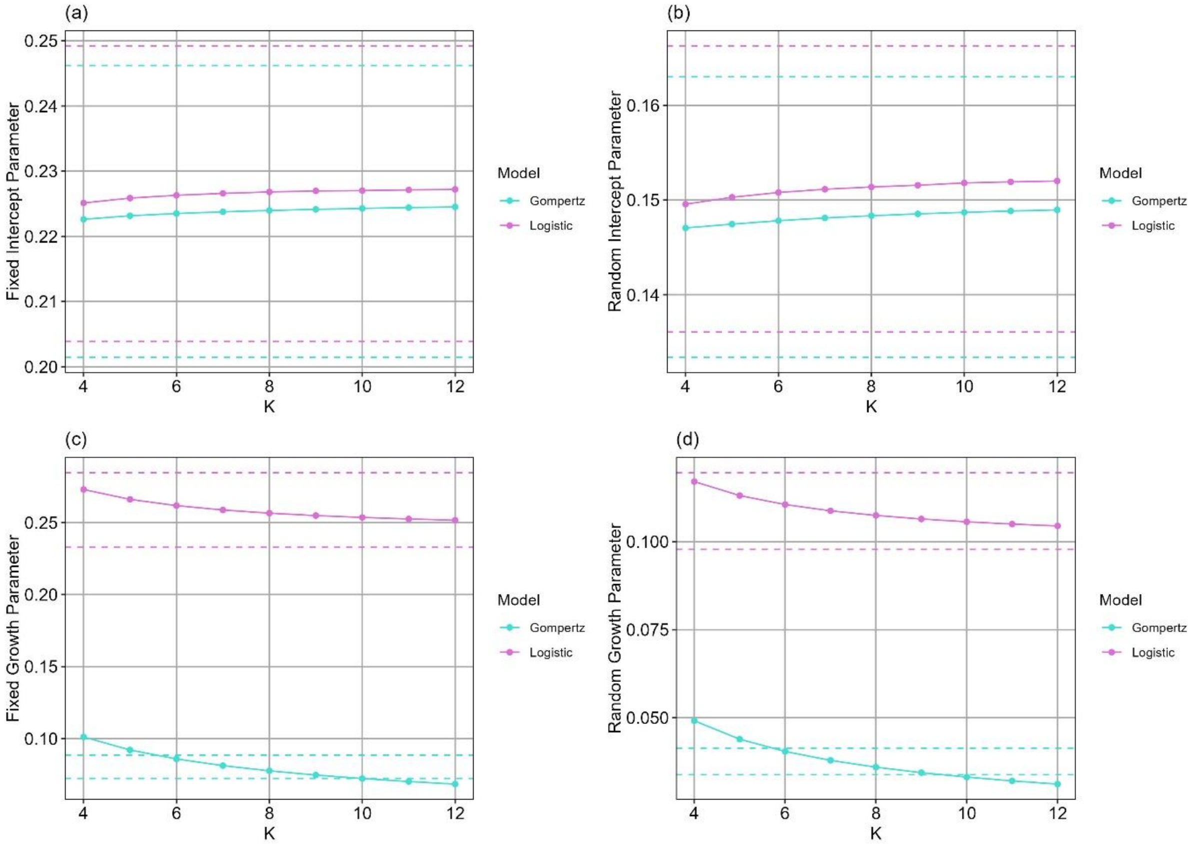

Figure S3.1: Change in model parameters for the Gompertz and logistic models with values of K = 4, …, 12. The dashed lines represent a +/- 10% boundary in parameter estimate from the mean. Model parameters include (a) fixed intercept; (b) random intercept; (c) fixed growth (i.e. population average growth rate); and (d) random growth (e.g. individual variation in growth rate).

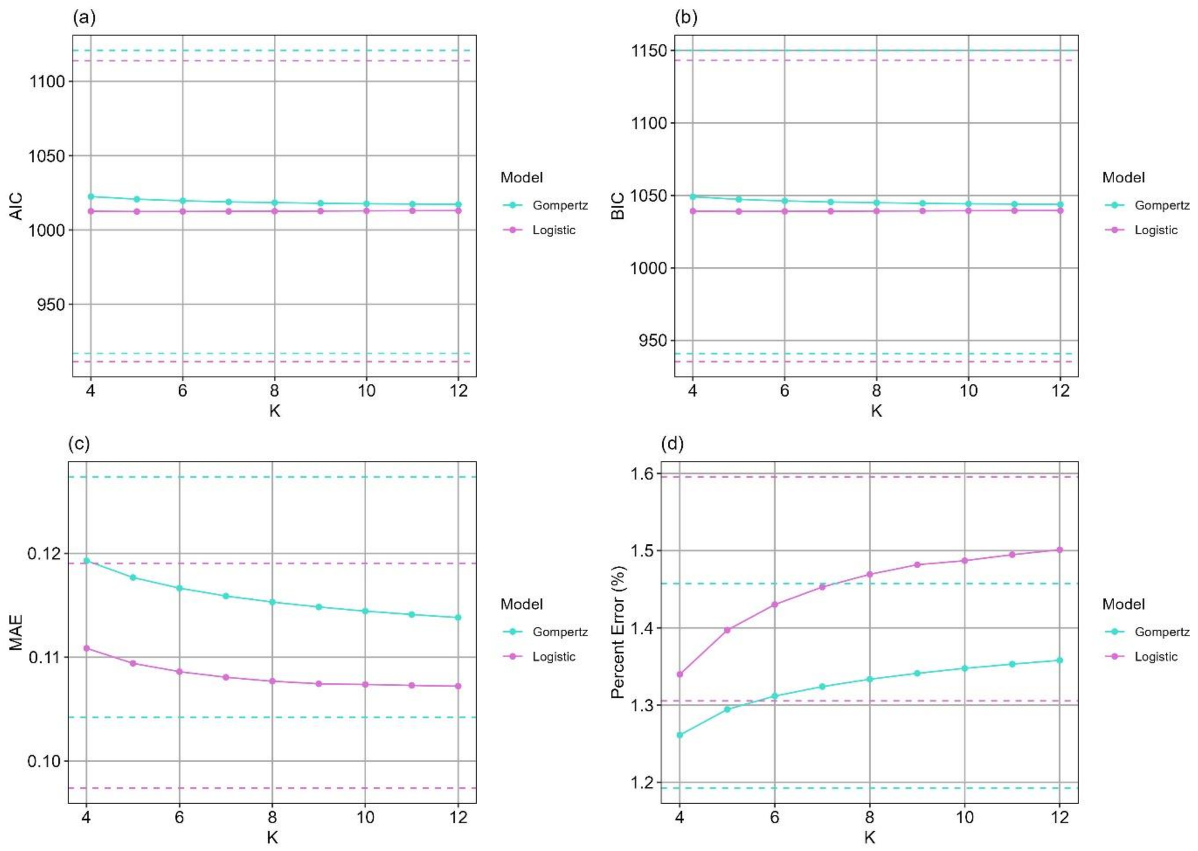

Figure S3.2: Change in model performance measures for the Gompertz and logistic models with values of K = 4, …, 12. The dashed lines represent a +/-10% boundary in parameter estimate from the mean. Model performance measures include (a) Akaike Information Criterion (AIC); (b) Bayesian Information Criterion (BIC); (c) mean absolute error (MAE) between the observed and predicted volumes over time; and (d) percentage error (%) between the observed and predicted volumes over time.

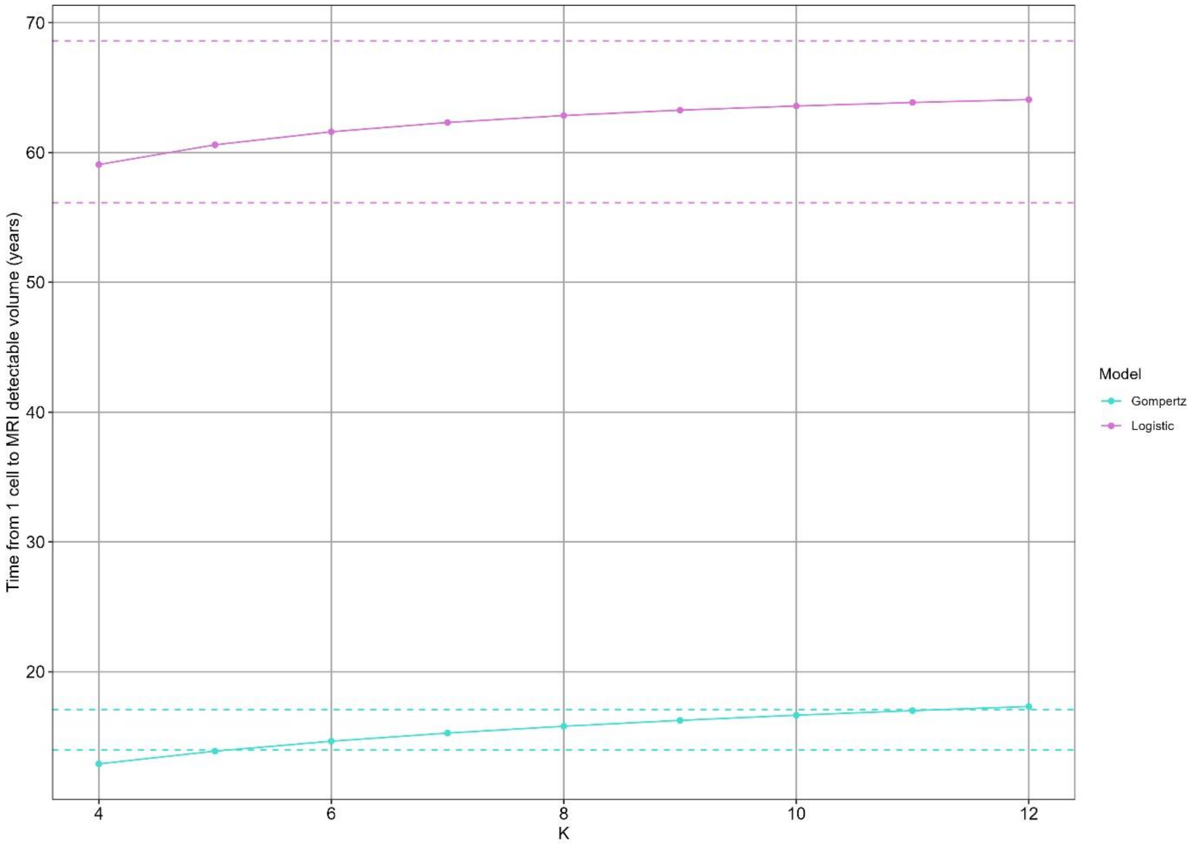

Figure S3.3: Change in estimated time for lesion to grow from 1 cell volume to an MRI detectable size for the Gompertz and logistic models with values of K = 4, …, 12. The dashed lines represent a +/-10% boundary in parameter estimate from the mean.

Supplementary 4: Model evaluation, calibration, and validation

Model evaluation

We tested the performance *of the exponential, Gompertz, and logistic mixed effects models when predicting all volumes, the first volume (models trained on measurements 2,…,n), and the final volume (models trained on measurements 1,…, n-1). We use AS patients with* ≥ 4 measurements to fit the models (n = 94).

Within the AS timeframe, three fixed effects models performed similarly (Figure S4.1 and Table S4.1). There was approximately a 20% error in volume prediction (Mean Absolute Error (MAE) ≈ 0.1 cm^3) across all time points for the three models. This rose to 29% when predicting the unseen final volume (MAE ≈ 0.25 cm^3) and 50% when back-predicting the unseen first volume (MAE ≈ 0.13 cm^3) for the exponential and Gompertz models. The logistic model failed to converge when trained the 94 individuals with ≥ 4 measurements to predict the first volume and the final volume.

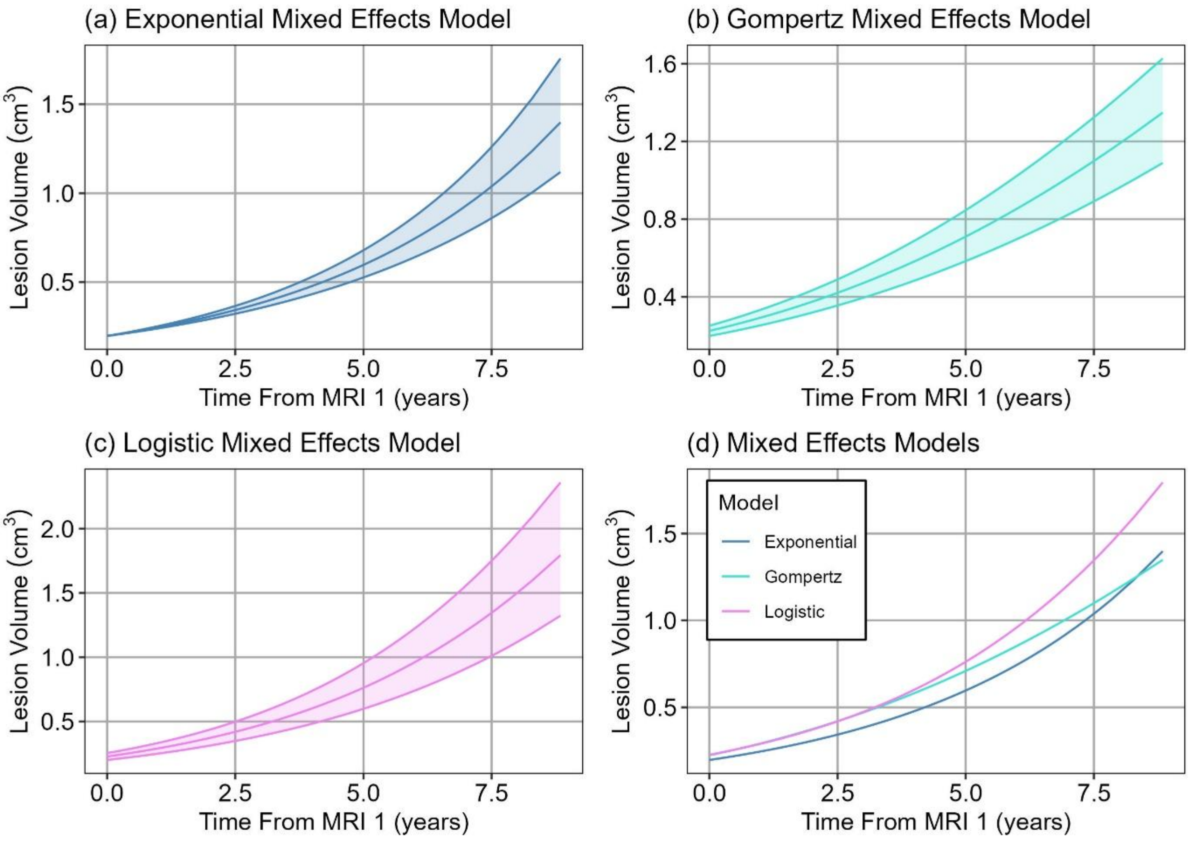

Figure S4.1: Fixed effects with 95% confidence interval for the (a) exponential; (b) Gompertz; and (c) logistic models. All three fixed effects models are shown in (d). The overall shape of the models are similar within the study timeframe, with the exponential model showing the slowest growth in the AS timeframe.

Table S4.1: Performance measures of the exponential, Gompertz, and logistic mixed effects models when predicting all volumes, the first volume (models trained on measurements 2,…,n), and the final volume (models trained on measurements 1,…, n-1).

	**Exponential**	**Gompertz**	**Logistic**
AIC	946	1010	1010
BIC	973	1040	1040
Predicting All Volumes			
MAE	0.091	0.113	0.106
% error	19.8	21.8	21.4
Predicting First Volume (n = 94)*[1]			
MAE	0.136	0.131	-*[2]
% error	51.2	47.8	-*[2]
Predicting Final Volume (n = 94)*[1]			
MAE	0.246	0.259	0.257
% error	29.2	29.0	30.0

*[1] Sample size is reduced to 94 individuals with ≥ 4 measurements when predicting the first and last volumes

*[2] Model would not converge

Akaike Information Criterion (AIC); Bayesian Information Criterion (BIC); mean absolute error (MAE); percentage error (% error)

Model Calibration

Figures S4.2 to S4.4 show calibration plots for the exponential, Gompertz, and logistic mixed effects models. Shown is the calibration when predicting all volumes in the dataset, predicting the first volume in the dataset when trained on volumes 2, …, n, and predicting the last volume in the dataset when trained on volumes 1, …, n-1. The logistic model did not converge when training on volumes 2, …, n or volumes 1, …, n-1, so calibration is only shown for predicting all volumes.

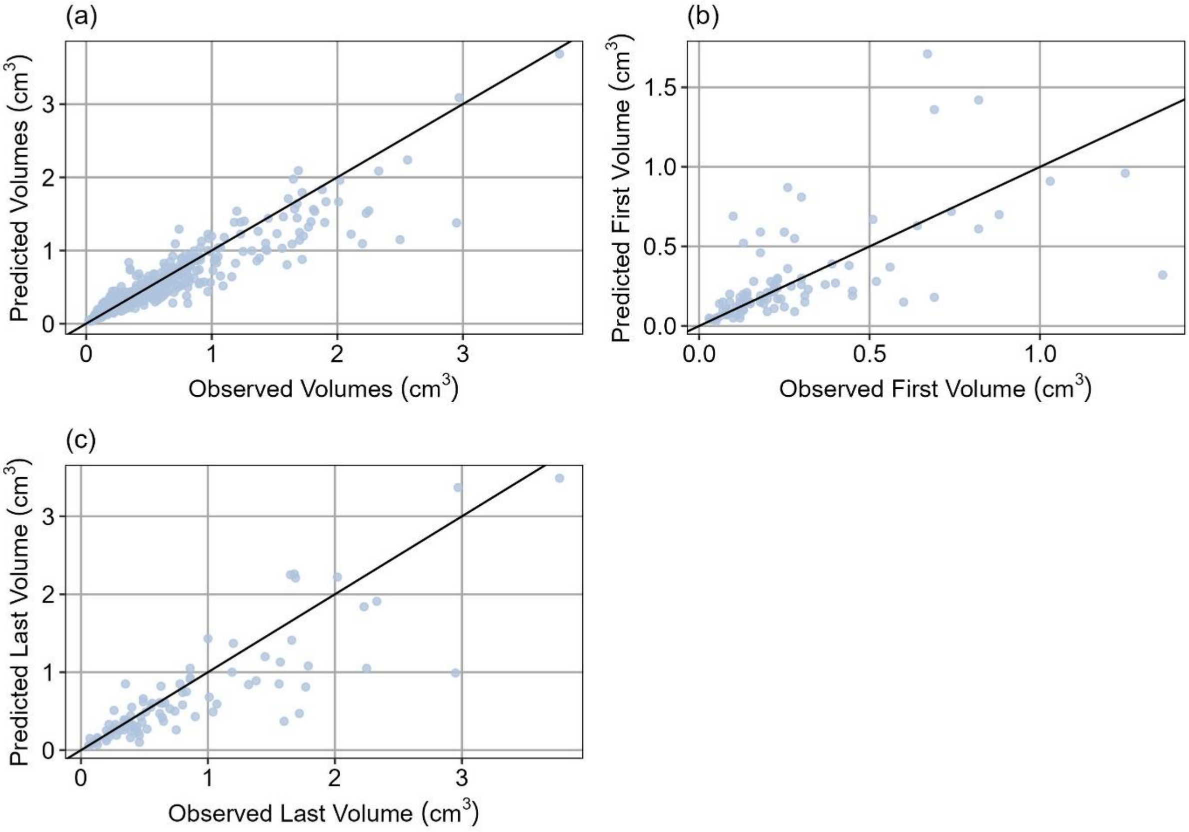

Figure S4.2: Calibration plots for the exponential model for predicting (a) all volumes in the dataset; (b) the first volume when trained on volumes 2, …, n; and (c) the final volume when trained on volumes 1, …, n-1.

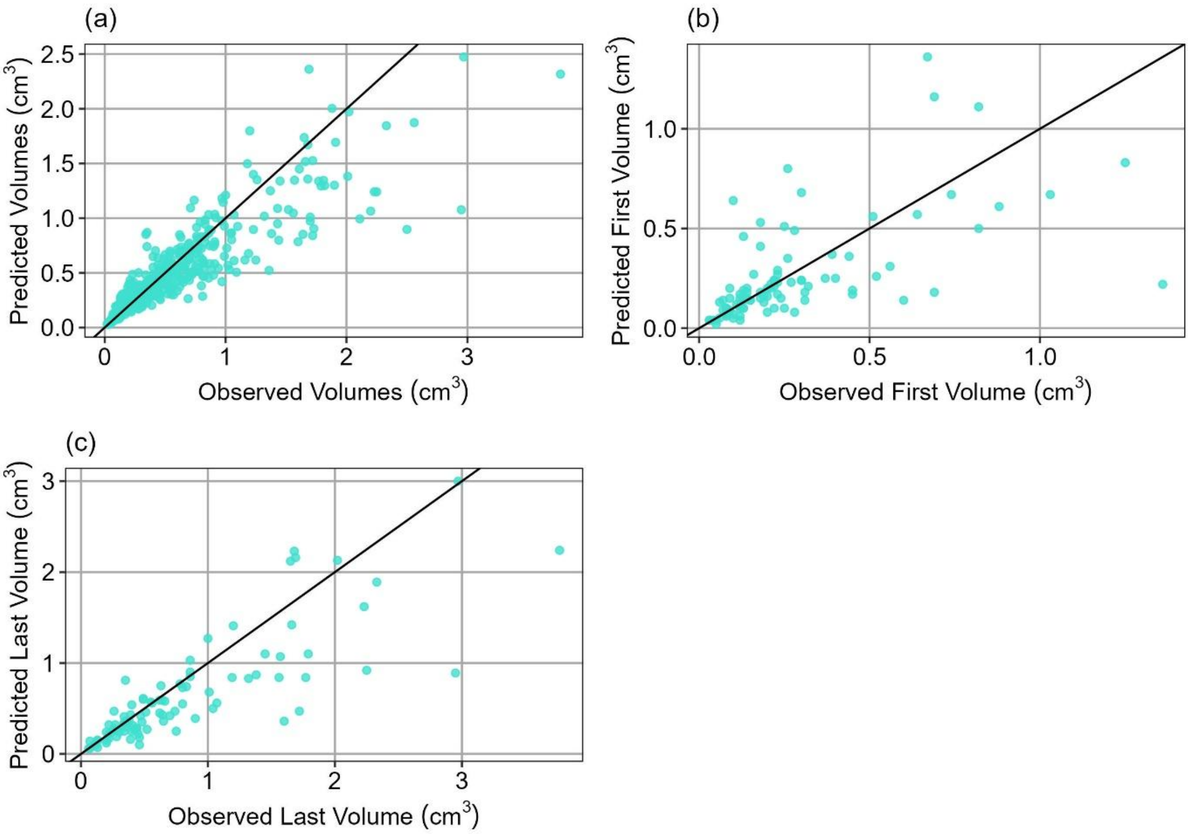

Figure S4.3: Calibration plots for the Gompertz model for predicting (a) all volumes in the dataset; (b) the first volume when trained on volumes 2, …, n; and (c) the final volume when trained on volumes 1, …, n-1.

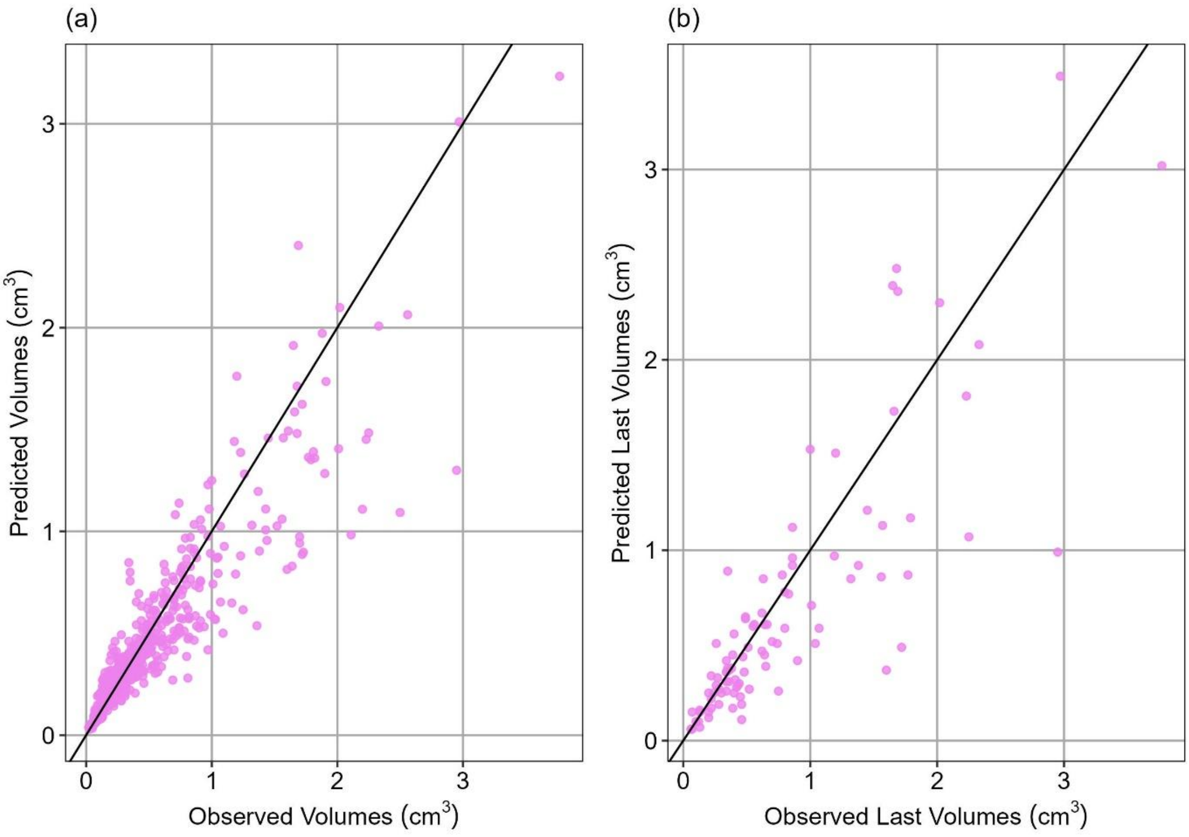

Figure S4.4: Calibration plots for the logistic model for predicting (a) all volumes in the dataset; and (b) the final volume when trained on volumes 1, …, n-1. The logistic model did not converge when training on volumes 2, …, n.

Model Validation

The exponential, Gompertz, and logistic models were validated by training on 75% of the data and then testing performance on the remaining unseen 25%. Volumes were predicted for each patient in the testing dataset at every time point using the population average growth rate.

The performance of all three models decreased when predicting volumes for unseen testing data (Table S4.2). The percentage errors were high, however, the volumes were relatively small. The exponential model had a slightly lower MAE and % error on the testing data compared to the Gompertz and logistic models. There was a large spread in performance throughout the data. Figures S4.5 to S4.7 show boxplots of the performance measures for the three models on the training and testing datasets, i.e. the mean absolute error (MAE) and the percent error (%) between the observed and predicted volumes.

Table S4.2: Validation of the exponential, Gompertz, and Logistic mixed effects models when trained on 75% of the data and tested on the remaining unseen 25%. The Mean Absolute Error (MAE) and Percentage Error (% Error) of the predicted volumes using the fixed effects model vs. the observed volumes for the training and testing datasets are detailed.

	**MAE**		**% Error**	
	Training	Testing	Training	Testing
**Exponential**	0.267	0.339	79.3	124
**Gompertz**	0.247	0.345	92.4	142
**Logistic**	0.280	0.355	95.9	150

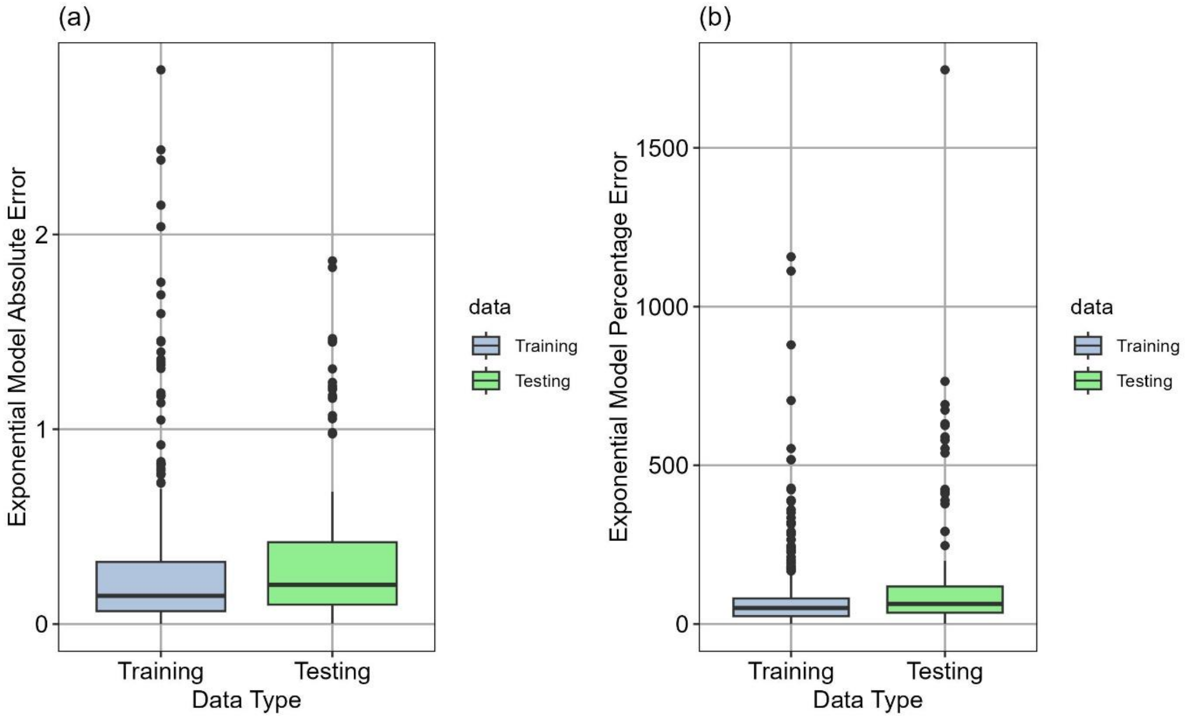

Figure S4.5: Boxplots of the (a) mean absolute error (MAE); and (b) the percent error (%) between the observed and predicted volumes for the exponential model. The plot shows the performance of the model on the 75% training dataset and the remaining unseen 25% testing dataset. Volumes were predicted for each patient in the sample at every time point using the population average growth rate.

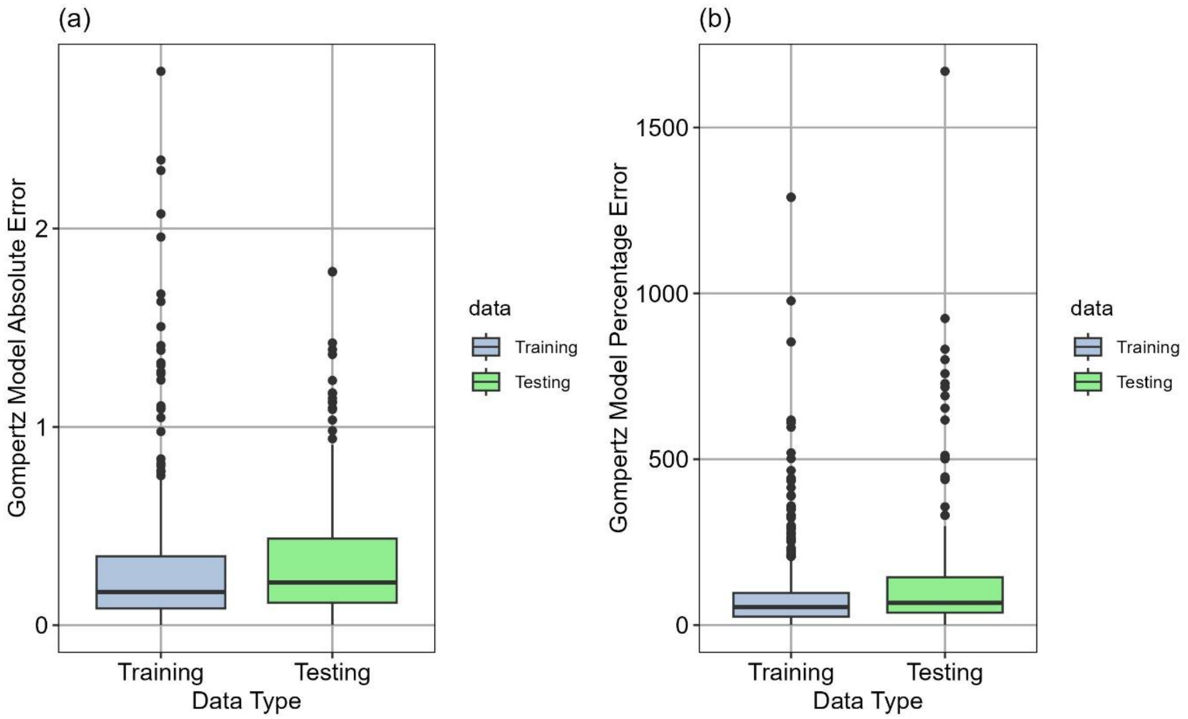

Figure S4.6: Boxplots of the (a) mean absolute error (MAE); and (b) the percent error (%) between the observed and predicted volumes for the Gompertz model. The plot shows the performance of the model on the 75% training dataset and the remaining unseen 25% testing dataset. Volumes were predicted for each patient in the sample at every time point using the population average growth rate.

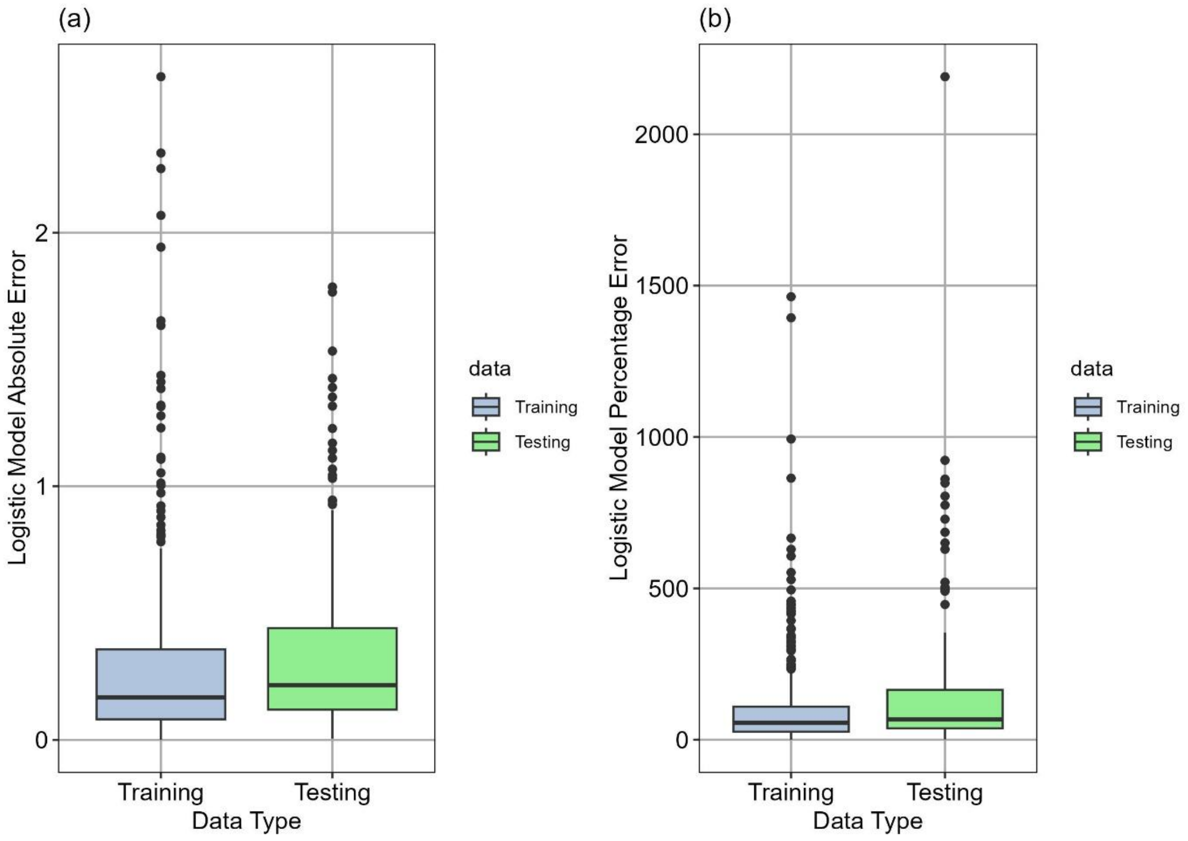

Figure S4.7: Boxplots of the (a) mean absolute error (MAE); and (b) the percent error (%) between the observed and predicted volumes for the logistic model. The plot shows the performance of the model on the 75% training dataset and the remaining unseen 25% testing dataset. Volumes were predicted for each patient in the sample at every time point using the population average growth rate.

Supplementary 5: Correlations between Gompertz growth rates and patient and tumour characteristics

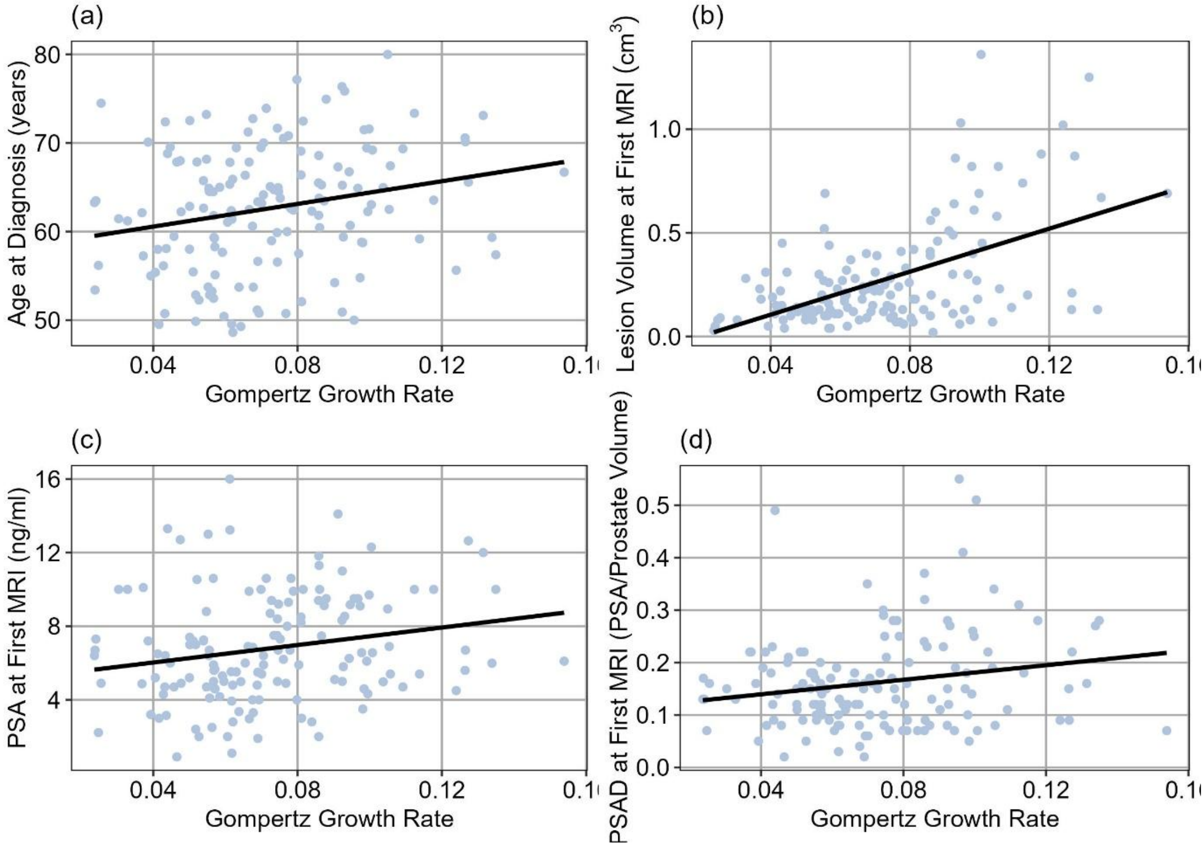

Figure S5.1: Scatter plots of the estimated individual lesion growth rates and (a) patient age at diagnosis ($R = 0.23$, $p < 0.01$); (b) lesion volume at first MRI ($R = 0.53$, $p < 0.01$); (c) PSA at first MRI ($R = 0.19$, $p = 0.02$); and (d) PSAD at first MRI ($R = 0.19$, $p = 0.02$). Growth rates are positively correlated with patient age, lesion volume, PSA level, and PSAD. The regression line is shown on each plot in black.
